# Green Light and Transcranial Direct Current Stimulation in Migraine Patients: A Preliminary Randomized Control Trial

**DOI:** 10.3390/brainsci15111209

**Published:** 2025-11-09

**Authors:** Amna Mahmood, Mirza Obaid Baig, Sumaiyah Obaid, Turki Abualait, Shahid Bashir

**Affiliations:** 1 Faculty of Rehabilitation & Allied Health Sciences, Riphah International University, Islamabad 44022, Pakistan; 2 College of Applied Medical Sciences, Imam Abdulrahman Bin Faisal University, Dammam 34212, Saudi Arabia; 3 Neuroscience Center, King Fahad Specialist Hospital, Dammam 32252, Saudi Arabia; 4 King Salman Center for Disability Research, Riyadh 11614, Saudi Arabia

**Keywords:** migraine, green light, transcranial direct current stimulation, pain

## Abstract

***Background:*** Migraine is a complex neurological condition characterized by a range of symptoms, such as intense to severe headaches, sensitivity to light and sound, and feelings of nausea and vomiting. The most common complaints regarding acute treatment are that medication causes adverse effects, that pain returns, or that pain relief is either too slow or inconsistent. Certain non-pharmacological methods, such as non-invasive neuromodulators, might be beneficial for alleviating migraines and require evidence for clinical judgment. ***Objective:*** The objective of the study was to determine the effects and compare the effects of green light and transcranial direct current stimulation on migraine frequency, intensity, impact, and quality of life. ***Methods:*** A randomized controlled trial was conducted with 69 migraine patients of both genders aged over 18 years, experiencing headache attacks lasting more than 4 h, characterized by pulsating and unilateral pain with an intensity of 5 or higher on the numeric pain scale. Active tDCS, sham tDCS, and green light, along with prescribed medications, were applied to Groups A, B, and C, respectively, for four weeks. The outcomes were measured at baseline, week 2, and week 4 for primary outcomes, including a structured headache diary, numeric pain scale, multidimensional pain inventory, and migraine-specific quality of life version 2.1 as a secondary variable. ***Results:*** Significant results were found for the NPS and MSQ with a *p*-value < 0.05 between and within the groups. There was a substantial reduction in pain intensity and improved quality of life in all three groups. Group A and Group C had *p*-values < 0.05 for most of the subscales of MPI, showing decreased pain interference, enhanced support, improved emotional function, and increased participation in everyday activities. Medication dependency in Group A was reduced to four weeks with 22 (95.7%) with ‘no medication’ use. The number of migraine attacks in Group A at four weeks reduced to ‘no attack’ for 7 (30.4%) participants, showing a reduction in both the number of attacks and their duration to 1–5 h in 12 (52.2%) participants. ***Conclusions:*** Both tDCS and green light therapy were found to be effective non-pharmacological therapies for reducing pain intensity, frequency, impact, and drug usage and for improving the quality of life of migraine patients. However, tDCS showed an advantage in terms of reducing pain intensity and its impact on daily living, while green light therapy showed a slightly greater improvement in quality of life.

## 1. Introduction

A widespread, chronic illness called migraine is usually characterized by repeated, incapacitating headache attacks and other symptoms, such as aura [1]. Migraine is a complex neurological condition characterized by a range of symptoms, such as intense to severe headaches, sensitivity to light and sound, and feelings of nausea and vomiting [2]. It currently holds the sixth position in terms of global disability among all neurological conditions [3] with the prevalence of migraines at 11.6% [4] and one-fifth (22.9%) of the Pakistani population reporting it [5]. Migraine can cause problems in relationships, the workplace, and educational endeavors. The basic mechanisms behind the premonitory, aura, headache, and postdrome phases of migraine are becoming increasingly clear to us [6].

Migraine patients often report lower quality of life, productivity at work, greater rates of missed work, increased use and abuse of over-the-counter and prescription drugs, including opioids, and increased use of healthcare services, including emergency room visits [7,8]. Treatments for migraines include both pharmaceutical approaches, such as preventive and acute drugs, and a range of non-pharmacological therapies [9]. Overuse of medication for headaches is prevalent and should be diagnosed, treated, and prevented when possible [10]. The most frequent grievances regarding acute treatment are that medication causes adverse effects, that pain returns, or that pain alleviation is either too slow or inconsistent [11]. Certain non-pharmacological methods, such as invasive and non-invasive neuromodulators, are currently in use [12] including transcranial direct current stimulation (tDCS), a technique that modifies brain excitability by applying milliamperes (mA) of electrical stimulation over the scalp. The majority of neurophysiological evidence actually supports M1 neuromodulation [13,14,15,16]. Resting membrane potential excitability is known to be modulated by tDCS, and this effect is known to endure for several hours or longer. Neurochemical or blood oxygen level-dependent (BOLD) signal modifications in various cortical or subcortical regions are examples of how this results in neuroplasticity [17]. Several studies have looked into the use of tDCS for migraine and reported it as a safe and efficacious treatment that significantly lowers headache frequency and pain severity [18].

Green light exposure has the ability to stimulate certain retinal pathways that play a role in the regulation of pain perception [19,20,21]. The retina, which is the part of the eye that is sensitive to light, contains specialized cells known as retinal ganglion cells. These cells are responsible for transmitting signals related to various functions, including the regulation of circadian rhythm and pain. By selectively activating these retinal ganglion cells, green light exposure can influence the perception of pain [22]. Intrinsically photosensitive retinal ganglion cells (ipRGCs) in the eyes of patients with migraine are hypersensitive and cause photosensitivity [23]. Cortical spreading depression (CSD) has been hypothesized to be the underlying mechanism of migraine with aura [24] and bright light irradiation is more likely to cause the CSD [23]. Encouraging outcomes show that exposure to green light dramatically decreased the intensity and duration of migraine headaches compared to white, blues and red lights [25].

Since green light exposure is a relatively new and innovative therapy option, there is a lack of data regarding its therapeutic effectiveness. To accurately confirm the effects of both treatment procedures, a larger population should be studied; the current research has been conducted on a limited sample size. Additionally, green light and tDCS have never been compared among migraine survivors. Therefore, the study aims to identify the effects as well as compare the effects of tDCS and green light on migraine sufferers.

## 2. Materials and Methods

A randomized controlled trial (RCT) with approval from the Research Ethical Committee of Riphah International University, Pakistan (Riphah/RCRAHS-ISB/REC/MS-PT/01726), registered at clinicaltrials.org (NCT06212869), was conducted in accordance with the CONSORT checklist and the Helsinki Declaration at Pakistan Railway General Hospital, Rawalpindi, Pakistan from 18 January 2024 to 31 July 2024. 

### 2.1. Participants

#### 2.1.1. Sample Size

The sample size was calculated using G*Power (Version 3.1 [26]) by setting the type I error α = 0.05 and type II error β = 0.2, using the F test of proportion. Results indicated that 12 participants in each arm would be sufficient to achieve 80% power to detect the effect of intervention (green light and tDCS) on migraine.

#### 2.1.2. Randomization

By using a sequentially numbered concealed opaque envelope (SNOSE) technique, a randomization sequence was generated with an allocation ratio of 1:1:1. Eighty-nine participants were randomly assigned in a 1:1:1 ratio to one of three groups: Group A received Anodal tDCS, Group B received sham tDCS, and Group C received green light. Participants included in the study were 18 years of age or older of any gender, meeting the International Headache Society’s diagnostic criteria (at least 5 episodes of pain lasting four to seventy-two hours (either untreated or unsuccessfully treated), with unilateral location, pulsating quality, vomiting and/or nausea during the headache or photophobia and phonophobia, and reporting a mean headache pain intensity of at least five on the numeric pain scale (NPS) in the ten weeks before enrollment. Participants with psychiatric disorders/mental illness, photophobia, presence of shunt and/or implant in the cranial region, brain tumors, or wound on the skull were excluded.

### 2.2. Intervention

#### 2.2.1. Green Light

Green light therapy was administered using a lamp emitting light at 525 ± 10 nm wavelength for one hour per day for four weeks in conjunction with prescribed medications. Patients were exposed to the light five days a week during the daytime in a peaceful environment, and were instructed not to fall asleep during treatment, keeping the therapy light within field of vision while engaging in activities like reading, exercising, listening to the Quran, and other such pursuits that did not require external light sources.

#### 2.2.2. Transcranial Direct Current Stimulation (tDCS)

Both sham and active tDCS were applied using the 1.1 Transcranial Electrical Stimulation device by Soterix^®^, Washington, DC, USA. The stimulation was delivered with the 35 cm^2^ electrodes for 15–20 min, three days a week during the daytime, at a rate of 1 mA, alongside prescribed medications [27]. The anodal electrode was placed at the left M1 area, identified by the 10/20 international EEG method, while the cathode was placed at the contralateral supraorbital region.

### 2.3. Outcome Measures

#### 2.3.1. Structured Headache Diary

The structured headache diary measures the primary outcome of frequency of headaches. To minimize recollection bias, patients at the headache clinic are required to maintain a headache diary for at least one month. By utilizing the headache diary, the diagnosis is strengthened by tracking the frequency, intensity, characteristics, and relevant contributing factors such as nutrition, stress, and the relationship between headaches and the menstrual cycle. Additionally, the frequency of analgesic usage is documented, which can provide information about treatment responses and potential of medication overdose according to the ICHD-2 amended criteria. A recent study has confirmed the effectiveness of the headache diaries for diagnosis. Therefore, keeping a headache diary aids in both diagnosis and treatment customization [27]. The validity of the structured headache diary ranges from 0.70 to 0.90 [28].

#### 2.3.2. Numeric Pain Scale (NPS)

The NPS assists clinicians in quickly measuring another primary outcome of pain intensity, leading to its global implementation in therapeutic practice. The NPS has a simple design, focusing on a scale of 0 to 10, where 0 represents “no pain” and 10 represents the “worst pain”. Hospitals commonly use the 0–10 NPS to quantify pain due to its convenience [29]. The internal coherence of the NPS range from 0.80 to 0.90 [28].

#### 2.3.3. Multidimensional Pain Inventory (MPI)

The MPI assesses the secondary outcome, the impact of migraine, through a 52-item, 12-scale questionnaire with three sections. The first part includes 5 items designed to evaluate significant aspects of living with chronic pain. Part II assesses perceptions of how their spouse or significant others respond to their pain behaviors. Part III evaluates patients’ participation in four types of daily activities. Additionally, a general activity scale score has been suggested for some applications [29]. The ICC value of the MPI is 0.70 to 0.90 [30].

#### 2.3.4. Migraine-Specific Quality of Life (MSQ 2.1)

The Migraine-Specific Quality of Life Questionnaire version 2.1 (MSQ 2.1) measures the secondary outcome, quality of life, using 14 items in three areas: Role Function-Restrictive (RFR), Role Function-Preventive (RFP), and Emotional Function (EF). RFR assesses how migraines affect everyday social and job-related tasks, RFP evaluates how migraines impact daily work and social activities, and EF measures the emotional impact of migraines [31]. The MSQ questionnaire contains the ICC value of 0.57 to 0.63 [32].

### 2.4. Data Collection

Initially, 115 patients meeting the eligibility criteria were screened, and 89 participants were included and randomly divided into three groups (Group A = 30, B = 29, and C = 30). After administering a pre-test to eligible individuals, data on the headache frequency, migraine intensity, migraine impact using the MPI, and quality of life were collected using a structural headache diary, numerical pain scale, MI, and MSQ 2.1, respectively.

**Figure 1:**
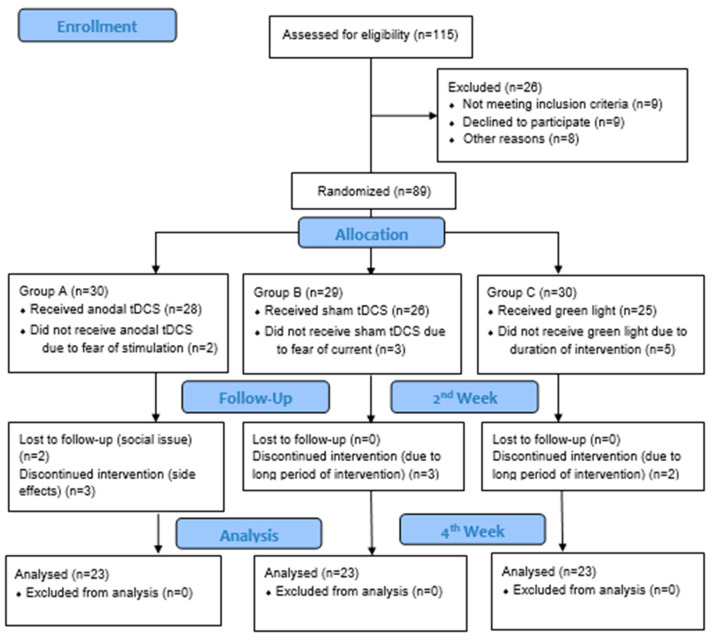
CONSORT diagram.

The participants in Group A received active tDCS, Group B received sham tDCS, and Group C received green light exposure. All participants were advised to continue taking the prescribed medications as usual. A total of 69 participants completed the study protocol and were included in the per protocol analysis (Figure 1).

### 2.5. Statistical Analysis

The data were analyzed using SPSS Version 21. Baseline characteristics were evaluated between the groups using the chi-square test for categorical variables and Kruskal–Wallis test for continuous variables due to the skewedness of data determined by the Kolmogorov–Smirnov test. The normality of the data was checked by using the Kolmogorov–Smirnov test, which indicated that the NPS, MPI, and MSQ were not normally distributed. Therefore, the Friedman test was used for within-group comparison, the Kruskal–Wallis test for between-group comparisons, and post hoc analysis was conducted using the Wilcoxon Signed Rank test. The level of significance was set at *p* value of less than 0.05. The effect size (partial η^2^) was calculated for the Kruskal–Wallis test using the formula η^2^ = (H − k + 1)/(n − k), where H is the Kruskal–Wallis statistic, k is the number of groups (3), and n is the sample size (69).

## 3. Results

### 3.1. Baseline Characteristics of Participants

The total number of participants was 69, with female dominance in Group A at 20 (28.9%), Group B at 14 (20.2%), and in Group C at 18 (26.0%). The average age of the participants in Groups A, B, and C was 24.6 ± 6.3, 23.8 ± 0.9, and 30.2 ± 11.8 years, respectively (See Table 1).

### 3.2. Structured Headache Diary Analysis

#### 3.2.1. Total Attacks

There was a significant reduction in migraine attacks per episode after exposure to the intervention, particularly in Group A. A large proportion of participants, especially in Groups A and C, did not experience any attacks by the end of week 4.

#### 3.2.2. Duration

There was a marked reduction in the duration of migraine episodes to zero, as most participants experienced no migraine attacks after the four weeks of intervention.

#### 3.2.3. Severity

After two weeks’ intervention, most participants reported not having had any attacks, and those who did experienced less severity. By the four-week mark, a higher percentage of individuals reported having light headaches or no episodes, continuing the trend of lower severity.

#### 3.2.4. Trigger Factors

Participants reported multiple triggering factors for their migraines including stress, exhaustion, and exposure to light. The results showed the influence of interventions on these triggering factors, with a decrease in headaches associated with stress observed in every group by the end of two weeks, while light and fatigue continued to be common triggers. Additionally, a larger decrease in stress as a trigger, especially for Group C, was found.

#### 3.2.5. Relieving Factors

Paracetamol was a popular way to relieve symptoms at baseline while after the four weeks of intervention; more participants found relief with bed rest instead of pharmacological agents.

### 3.3. Medication Dependency

Most participants in all groups used paracetamol to manage their migraine attacks which was reduced to no medication after four weeks, showing a significant impact of the intervention in all three groups.

### 3.4. Between-Group Analysis Across Different Outcome Measures

#### 3.4.1. Numeric Pain Scale (NPS)

There were significant effects of interventions at two and four weeks with a *p*-value < 0.001 (see Table 2).

#### 3.4.2. Multidimensional Pain Inventory (MPI)

There were marked significant effects of interventions among the groups after four weeks with the *p* > 0.05 in the A1 (patient perceived interference of pain in various areas of functioning), A2 (patient perception of support received by others), A3 (level of pain severity), A4 (patient perception of control), A5 (level of effective distress), B2 (patient perceived frequency of solicitous response), B3 (patient perceived frequency of distracting response), C1 (performance of household chores), C2 (performance of outdoor work), C3 (performance of activities away from home), and C4 (performance of social activities) (See Table 2).

#### 3.4.3. Migraine-Specific Quality of Life (MSQ)

The interventions showed significant effects on the MSQ total after interventions of two and four weeks (See Table 2).

### 3.5. Within-Group Analysis Across Different Outcome Measures

#### 3.5.1. Numeric Pain Scale (NPS)

For the variable NPS, Groups A, B, and C showed significant results within the group with a *p*-value < 0.05, indicating that all three groups had effects on the reduction in pain intensity (See Table 3).

From baseline to four weeks, Group A’s pain intensity consistently and significantly decreased, with persistent decreases between two and four weeks. Group B showed considerable reductions from baseline to four weeks, with persistent reductions between two weeks and four weeks, but initially showed no improvement. Group C showed a significant decrease in pain severity in all time frames.

#### 3.5.2. Multidimensional Pain Inventory (MPI)

For variables A1, A3, A4, and A5, Groups A, B, and C showed *p*-values < 0.05, indicating significant results within the group. For variable A2, Group A and Group B had a *p*-value > 0.05, showing non-significant results within the group. However, Group C had a *p*-value < 0.05, which shows significant results within the group (See Table 3).

For variable B1, Group A, Group B, and Group C had a *p*-value > 0.05, showing non-significant results within the group. For variables B2 and B3, Group A and Group C had a *p*-value < 0.05, indicating significant results within the group. However, Group B had a *p*-value > 0.05, showing non-significant results within the group (See Table 3).

For variables C1, C2, and C3, Groups A, B, and C had *p*-values < 0.05, indicating significant results within the group. For variable C4, Group A and Group C had a *p*-value < 0.05, indicating significant results within the group. However, Group B had a *p*-value > 0.05, showing non-significant results within the group (See Table 3).

From baseline to two weeks and four weeks, Group A showed significant improvements in most MPI subscales, indicating decreased pain interference, enhanced support, and improved emotional function. Group B also showed notable progress, especially from baseline to the 4-week mark, indicating improved life management and decreased affective distress. The MPI subscales significantly improved for Group C, indicating a decrease in the impact of pain and an increase in participation in everyday activities.

#### 3.5.3. Migraine-Specific Quality of Life

For the variable MSQ total, Groups A, B, and C showed a *p*-value < 0.05, which shows significant results within the group, denoting that all three groups had an impact on improving quality of life (See Table 3).

Group A showed a significant increase across all time frames of MSQ, indicating persistent improvement in quality of life. Group B showed significant improvement across the baseline to four-week time frame, showing better quality of life. Some improvement is also seen from baseline to two weeks and from two weeks to four weeks. In all time frames, Group C showed a significant increase in the MSQ, indicating a better quality of life.

## 4. Discussion

The focus of the present study was to determine the effects of green light and tDCS in migraine patients, as well as to compare both interventions. The current study findings indicate that the majority of participants were females. The mean ages of Group A, Group B, and Group C were 24.6 ± 6.3, 23.8 ± 0.9, and 30.2 ± 11.8, respectively, with right-handed dominance among the majority of participants.

The results of the current study suggest that within the observed time frames, all three groups (A, B, and C) demonstrate noteworthy gains in various areas linked to migraine treatment. Group A continuously showed significant improvements in every variable, indicating a very favorable reaction to the intervention. Group B exhibited gradual but significant gains over time, especially in terms of quality of life and pain severity. Similarly, Group A and Group C consistently showed notable improvements in all variables and time periods.

The current study results show that significant differences were observed between the group analyses of the Numeric Pain Scale, Multidimensional Pain Inventory, and Migraine-Specific Quality of Life version 2.1 after two and four weeks of intervention. Within-group analysis of the Numeric Pain Scale, Multidimensional Pain Inventory, and Migraine-Specific Quality of Life version 2.1 shows significant results after intervention. The results indicate that the use of NPS, MPI, and MSQ was effective for migraine patients.

In a study conducted in 2020 by Martin et al., participants’ quality of life was assessed using the EQ5D-5L, the Modified University of Arizona Pain Clinic Follow-up Questionnaire was utilized to measure changes in pain intensity following treatment, and the Headache Impact Test-6 (HIT-6) was used to measure functional capacity. The results showed that exposure to green light-emitting diodes produced a statistically significant reduction in NPS scores, HIT-6 scores in all migraine groups, and in the number of headache days per month in both chronic and episodic migraine groups. Moreover, green light significantly improved the quality of life (EQ-5D-5L) and pain perception (Modified University of Arizona Pain Clinic Follow-up Questionnaire) among people with episodic, chronic, and mixed migraines [2]. Similarly to the current study, green light showed a significant reduction in pain intensity (NPS), frequency (structured headache diary), impact (MPI), and improvement in quality of life (MSQ) in migraine patients.

The current study has shown positive results in the reduction in pain intensity, frequency, impact, and quality of life in both the tDCS and sham treatment groups. In contrast, a 2020 study found that patients benefitted from tDCS in terms of migraine episode frequency, number of headache days, attack duration, and pain severity compared to those in the sham treatment group [33].

According to a 2017 study utilizing the Medical Outcomes Study 36-Item Short-Form Health Survey (SF-36) for quality of life, the Visual Analogue Scale (VAS) for pain intensity, and the HIT-6 for impact of headache, the DLPFC group showed reductions in all aspects in tDCS [34]. On the other hand, the current study used the NPS for pain intensity, a structured headache diary for frequency, the MPI for impact, and the MSQ, showing significant results in all aspects with tDCS.

In a 2016 study, the VAS showed that green light has a positive impact in decreasing pain intensity compared to white, blue, amber, and red lights [23]. However, in the current study, “Green Light and tDCS in migraine patients”, the NPS showed that green light has a significant impact on reducing pain intensity.

The current study utilized MSQ to determine the quality of life of migraine patients. Recent study results show a significant decrease in intensity, frequency, and medication usage in both active and sham tDCS groups. Additionally, research conducted in 2021 by Sirin et al. showed that the Migraine Disability Assessment Test (MIDAS) and Beck Depression Scale (BDS) were used to assess the quality of life of patients. The results indicated a significant decrease in migraine attack frequency, number of headache days, duration of attacks, and symptomatic analgesic drug use in patients receiving active tDCS, compared to the control group [35].

A previous study found that daily pain measured by the VAS, length of migraine episodes, Patient Global Assessment (PGA), and Clinical Global Impression (CGI) were used to gather relevant information. The Mini-Mental State Examination (MMSE) and Digit Span (forward and backward) were also used to assess cognitive function. The results showed a significant difference in pain intensity and length of migraine episodes only in the active tDCS group after a four-week follow-up [36]. In the current study, NPS showed a significant difference in pain intensity and frequency in both the active and sham tDCS groups after four weeks of follow-up.

A study used the NPS as a significant and valid tool for determining pain intensity [37]. After four weeks of treatment, a significant difference was shown in the reduction in pain intensity and drug usage. The VAS was shown to be useful in measuring pain severity in a prior study. After four and eight weeks of therapy, the results demonstrated a statistically significant decrease in the frequency of attacks and medications that were stopped. At weeks 4, 8, and 12, there was a statistically significant decrease in the level of pain. A study from 2020 indicates that headache severity, frequency, and duration significantly reduced and were measured with tools (VAS, HIT-6, MPI, and headache diary). The quality of life was measured with MSQOL in a meta-analysis study [38]. The current study indicates that headache intensity, frequency, and impact were significantly reduced and measured with tools (NPS, structured headache diary, MPI), and quality of life improvement was measured by the MSQ. This study was an RCT. The results of the current study showed that NPS has an impact on reducing pain intensity, a structured headache diary has an impact on reducing headache frequency, and medications were stopped with anodal stimulation. A study from 2019 showed that the VAS had an impact on the average number of migraine-free days, a reduction in tablet usage, and that discomfort increased significantly with cathodal stimulation after therapy [39].

A recent study from 2020 utilized the Migraine Screen Questionnaire (MS-Q) and headache diary tools. The results showed a significant reduction in frequency, duration, and intensity of migraine pain in both experimental groups compared to the sham intervention group at the post-test and follow-up [40]. The current study used NPS, MPI, structured headache diary, and MSQ as tools showing significant differences in the reduction in pain intensity, headache frequency, impact, and improvement in quality of life in both the experimental and control groups at post-test.

The recent study of four weeks of intervention indicates significant results in pain intensity, frequency, drug usage, and impact decrease and improvement in quality of life in the active tDCS group, similar to the sham tDCS group. However, a prior study from 2022 showed that the numerical rating scale was used to assess the duration and pain intensity, HIT-6 was used to assess the impact of headache on the overall quality of life, MIDAS was used to assess the number of days of disability due to migraine, and HADS was used to assess symptoms of anxiety and depression. The results of the study showed that a reduction in the average number of migraine attacks per month was observed. The results also showed that the reduction in the monthly number of migraine attacks was significantly higher in the active group than in the sham group [41].

Some limitations within the current study should be considered. First, findings failed to describe the mechanism of action as no objective data/tools were incorporated to understand the underlying activity of the brain. Second, the sham stimulation showed significant improvement in all variables, making it difficult to determine the true intervention results. Additionally, there was no blinding planned in the study; hence, the results cannot be free from bias. Additionally, the details of green light exposure were not considered, including the distance from the light source to the participants, the size of the room, illuminance, etc., which may influence the outcome. Lastly, this study was designed as a preliminary trial with a relatively small sample size, short-term follow-up, and without blinding of outcome assessors. While the results provide initial support for the potential efficacy of tDCS and green light exposure in migraine management, further large-scale, multicenter randomized controlled trials are needed to confirm these findings and evaluate long-term outcomes.

## 5. Conclusions

Both tDCS and green light therapy were found to be effective non-pharmacological therapies for the reduction in pain intensity, frequency, and impact, and improving the QoL of migraine patients. However, tDCS showed an advantage in reducing pain intensity and its impact on daily living, whereas green light therapy showed improvement in quality of life. Additionally, both therapies affect the drug/pharmacological usage for addressing migraine attacks. These results validate the application of both therapies as practical approaches to migraine treatment. Future studies utilizing brain imaging combined with neurophysiological techniques such as electroencephalograph (EEG) would shed light on the neural bases and the brain activities underlying the effect of green light exposure in patients with migraine.

## Figures and Tables

**Table 1:** Baseline demographic characteristics of participants.

Variables		Group A	Group B	Group C	*p*-Value
**Age (years)**		24.6 ± 6.3	23.8 ± 0.9	30.2 ± 11.8	0.001
**Gender**	Male	3 (13.04%)	9 (39.13%)	5 (21.73%)	0.368
	Female	20 (86.96%)	14 (60.87%)	18 (78.27%)	
**Occupation**	Doctor	20 (86.96%)	22 (95.66%)	14 (60.86%)	0.003
	Engineer	1 (4.34%)	0	2 (8.69%)	
	Labor	0	1 (4.34%)	2 (8.69%)	
	Student	2 (8.69%)	0	1 (4.34%)	
	Housewife	0	0	4 (17.39%)	
**Educational status**	Intermediate	2 (8.69%)	0	1 (4.34%)	0.080
	Undergraduate	17 (73.91%)	17 (73.91%)	12 (52.17%)	
	Postgraduate	4 (17.39%)	6 (26.08%)	10 (43.47%)	
**Site of involvement**	Frontal	4 (17.39%)	8 (34.78%)	6 (26.08%)	0.219
	Temporal	6 (26.08%)	1 (4.34%)	1 (4.34%)	
	Parietal	4 (17.39%)	4 (17.39%)	1 (4.34%)	
	Occipital	4 (17.39%)	2 (8.69%)	3 (13.04%)	
	F + T	2 (8.69%)	3 (13.04%)	2 (8.69%)	
	F + P	2 (8.69%)	0	4 (17.39%)	
	T + P	0	2 (8.69%)	2 (8.69%)	
	T + O	0	0	2 (8.69%)	
	P + O	1 (4.34%)	3 (13.04%)	2 (8.69%)	
**Migraine duration**	1–5 h	13 (56.52%)	6 (26.08%)	11 (47.82%)	0.500
	6–10 h	2 (8.69%)	10 (43.47%)	4 (17.39%)	
	21–25 h	6 (26.08%)	4 (17.39%)	6 (26.08%)	
	2 days	2 (8.69%)	3 (13.04%)	2 (8.69%)	
**Comorbid**	Systemic issue	5 (21.73%)	3 (13.04%)	5 (21.73%)	0.001
	MSK issues	4 (17.39%)	0	2 (8.69%)	
	CNS issues	2 (8.69%)	0	0	
	Nothing	12 (52.17%)	20 (86.95%)	16 (69.56%)	
**Dominant side**	Right-handers	21 (91.30%)	21 (91.30%)	22 (95.66%)	0.351
	Left-handers	0	2 (8.69%)	1 (4.34%)	
	Mixed-handers	2 (8.69%)	0	0	
**Diet**	Normal	8 (34.78%)	10 (43.47%)	14 (60.86%)	0.197
	Skip meal	5 (21.73%)	5 (21.73%)	3 (13.04%)	
	Excessive use of food	10 (43.47%)	8 (34.78%)	6 (26.08%)	
**Physical activity**	Sedentary lifestyle	6 (26.08%)	4 (17.39%)	4 (17.39%)	0.914
	Active lifestyle	8 (34.78%)	11 (47.8%)	15 (65.21%)	
	Walk daily	7 (30.43%)	8 (34.78%)	4 (17.39%)	
	Exercise daily	2 (8.69%)	0	0	
**BMI**	Underweight	2 (8.69%)	3 (13.04%)	1 (4.34%)	0.596
	Normal	14 (60.86%)	14 (60.86%)	12 (52.17%)	
	Overweight	3 (13.04%)	2 (8.69%)	8 (34.78%)	
	Obese	4 (17.39%)	4 (17.39%)	2 (8.69%)	

BMI = body mass index, MSK = musculoskeletal, CNS = central nervous system, F = frontal, T = temporal, P = parietal, O = occipital.

**Table 2:** Between-group analysis.

	Time	Groups	Mean Rank	*p*-Value	Effect Size (η^2^)
Numeric pain scale	Baseline	Group A	32.52	0.749	
		Group B	35.87		
		Group C	36.61		
	2 weeks	Group A	22.63	<0.001	0.730
		Group B	52.43		
		Group C	29.93		
	4 weeks	Group A	20.98	<0.001	0.706
		Group B	58		
		Group C	26.02		
Multidimensional pain inventory					
A1 Interference of pain	Baseline	Group A	35.48	0.978	
		Group B	34.35		
		Group C	35.17		
	2 weeks	Group A	25.83	<0.001	0.35
		Group B	53.30		
		Group C	25.87		
	4 weeks	Group A	23.85	<0.001	0.52
		Group B	57.65		
		Group C	23.50		
A2 Support received by other	Baseline	Group A	32.07	0.051	
		Group B	41.59		
		Group C	31.35		
	2 weeks	Group A	33.30	0.005	0.09
		Group B	43.09		
		Group C	28.61		
	4 weeks	Group A	33.37	<0.001	0.17
		Group B	44.13		
		Group C	27.50		
A3 Pain severity	Baseline	Group A	36.87	0.649	
		Group B	32.09		
		Group C	36.04		
	2 weeks	Group A	29.35	<0.001	0.41
		Group B	48.74		
		Group C	26.91		
	4 weeks	Group A	26.96	<0.001	0.61
		Group B	55.80		
		Group C	22.24		
A4 Control perception	Baseline	Group A	35.13	0.340	
		Group B	39.09		
		Group C	30.78		
	2 weeks	Group A	44.70	<0.001	0.56
		Group B	20.78		
		Group C	39.52		
	4 weeks	Group A	45.83	<0.001	0.63
		Group B	12.65		
		Group C	46.52		
A5 Effective distress	Baseline	Group A	41.28	1.0	
		Group B	26.37		
		Group C	37.35		
	2 weeks	Group A	31.11	0.022	0.58
		Group B	44.02		
		Group C	29.87		
	4 weeks	Group A	26.24	<0.001	0.79
		Group B	53.72		
		Group C	25.04		
B1 Punishing response	Baseline	Group A	35.43	0.372	
		Group B	33.91		
		Group C	35.65		
	2 weeks	Group A	37	0.334	0.02
		Group B	32.50		
		Group C	35.50		
	4 weeks	Group A	37	0.334	0.02
		Group B	32.50		
		Group C	35.50		
B2 Solicitous response	Baseline	Group A	34.50	0.520	
		Group B	37.50		
		Group C	33		
	2 weeks	Group A	32.33	0.181	0.00
		Group B	38.93		
		Group C	33.74		
	4 weeks	Group A	35.83	0.017	0.06
		Group B	40.17		
		Group C	31		
B3 Distracting response	Baseline	Group A	34.02	0.472	
		Group B	38.78		
		Group C	32.20		
	2 weeks	Group A	32.22	0.036	0.03
		Group B	43.04		
		Group C	29.74		
	4 weeks	Group A	31.93	<0.001	0.17
		Group B	46.35		
		Group C	26.72		
C1 Household chores	Baseline	Group A	36.00	0.131	
		Group B	36.00		
		Group C	33.00		
	2 weeks	Group A	27.20	<0.001	0.55
		Group B	55.13		
		Group C	22.67		
	4 weeks	Group A	24.86	<0.001	0.85
		Group B	55.61		
		Group C	22.50		
C2 Outdoor work	Baseline	Group A	36.67	0.875	
		Group B	34.11		
		Group C	34.22		
	2 weeks	Group A	45.30	<0.001	0.32
		Group B	18.96		
		Group C	40.74		
	4 weeks	Group A	48.98	<0.001	0.35
		Group B	12.54		
		Group C	43.48		
C3 Activities away from home	Baseline	Group A	29.22	0.193	
		Group B	37.15		
		Group C	38.63		
	2 weeks	Group A	38.91	<0.001	0.58
		Group B	19.13		
		Group C	46.96		
	4 weeks	Group A	44.65	<0.001	0.92
		Group B	12		
		Group C	48.35		
C4 Social activities	Baseline	Group A	35.22	0.418	
		Group B	31.13		
		Group C	38.65		
	2 weeks	Group A	42.48	<0.001	0.64
		Group B	16.15		
		Group C	46.37		
	4 weeks	Group A	42.02	<0.001	0.76
		Group B	12.98		
		Group C	48.00		
Migraine-specific quality of life					
Domain 1 Restrictive	Baseline	Group A	36.50	0.470	
		Group B	36.50		
		Group C	32		
	2 weeks	Group A	22.89	<0.001	0.52
		Group B	55.22		
		Group C	26.89		
	4 weeks	Group A	23.07	<0.001	0.64
		Group B	57.35		
		Group C	24.59		
Domain 2 Preventive	Baseline	Group A	37.28	0.236	
		Group B	29.63		
		Group C	38.09		
	2 weeks	Group A	23.91	<0.001	0.381
		Group B	52		
		Group C	29.09		
	4 weeks	Group A	23.50	<0.001	0.60
		Group B	56.57		
		Group C	24.93		
Domain 3 Emotional function	Baseline	Group A	35.02	0.847	
		Group B	36.57		
		Group C	33.41		
	2 weeks	Group A	26.30	<0.001	0.45
		Group B	54.17		
		Group C	24.52		
	4 weeks	Group A	22.35	<0.001	0.60
		Group B	56.83		
		Group C	25.83		
MSQ total	Baseline	Group A	35.07	0.876	
		Group B	33.65		
		Group C	36.28		
	2 weeks	Group A	21.50	<0.001	0.59
		Group B	56.17		
		Group C	27.33		
	4 weeks	Group A	22.50	<0.001	0.70
		Group B	58		
		Group C	24.50		

**Table 3:** Within-group analysis.

Variable	Time	Group A			Group B			Group C		
		Median (IQR)	Mean Rank	*p*-Value	Median (IQR)	Mean Rank	*p*-Value	Median (IQR)	Mean Rank	*p*-Value
NPS	Baseline	4(0)	3	<0.001	4(0)	2.30	<0.001	4(0)	3.48	<0.001
	2 weeks	3(1)	2		4(0)	2.30		2(1)	1.96	
	4 weeks	1(1)	1		3(1)	1.39		1(1)	1.04	
Multidimensional pain inventory										
A1	Baseline	1(0)	1.9	<0.001	1(0)	1.48	<0.001	1(0)	1.04	<0.001
	2 weeks	2(1)	2.17		1(1)	1.93		2(1)	2.17	
	4 weeks	3(0)	2.74		2(1)	2.59		3(0)	2.78	
A2	Baseline	1(0)	2.54	0.392	1(2)	2.95	0.112	1(0)	2.67	0.009
	2 weeks	1(0)	2.48		1(2)	2.41		1(0)	2.37	
	4 weeks	1(0)	2.43		1(2)	2.41		1(0)	2.28	
A3	Baseline	1(0)	1.50	<0.001	1(0)	1.87	<0.001	1(0)	1.50	<0.001
	2 weeks	3(1)	3.30		2(1)	2.78		3(1)	3.33	
	4 weeks	3(0)	3.70		2(0)	3.48		3(0)	3.67	
A4	Baseline	3(0)	3.37	<0.001	3(0)	2.87	<0.001	3(0)	3.43	<0.001
	2 weeks	2(1)	1.78		3(0)	2.43		1(1)	1.72	
	4 weeks	1(0)	1.48		2(1)	1.83		1(0)	1.41	
A5	Baseline	1(0)	1.50	<0.001	1(0)	2.09	<0.001	1(0)	1.50	<0.001
	2 weeks	2(1)	3.26		1(0)	2.43		2(1)	3.26	
	4 weeks	3(0)	3.74		2(1)	3.39		3(0)	3.74	
B1	Baseline	3(0)	2.54	0.112	3(0)	2	1.0	3(0)	1.96	0.368
	2 weeks	3(0)	2.54		3(0)	2		3(0)	2.02	
	4 weeks	3(0)	2.54		3(0)	2		3(0)	2.02	
B2	Baseline	1(0)	2.17	0.018	1(2)	2.09	0.135	1(0)	2.13	0.050
	2 weeks	1(0)	1.91		1(1)	1.96		1(0)	2	
	4 weeks	1(0)	1.91		1(1)	1.96		1(0)	1.87	
B3	Baseline	2(2)	2.41	<0.001	2(2)	2.04	0.135	2(2)	2.41	<0.001
	2 weeks	1(1)	2.02		2(2)	2.04		1(1)	2.02	
	4 weeks	1(1)	1.57		2(2)	1.91		1(0)	1.57	
C1	Baseline	3(0)	3	<0.001	3(0)	2.33	0.003	3(0)	3	<0.001
	2 weeks	2(1)	1.74		3(1)	2.93		1(1)	1.70	
	4 weeks	1(0)	1.26		3(1)	1.74		1(0)	1.30	
C2	Baseline	3(0)	2.96	<0.001	3(0)	2.54	<0.001	3(0)	3	<0.001
	2 weeks	2(0)	1.80		3(1)	1.91		2(1)	1.74	
	4 weeks	1(1)	1.24		2(1)	1.54		1(0)	1.26	
C3	Baseline	3(0)	3	<0.001	3(0)	2.33	0.001	3(0)	3	<0.001
	2 weeks	1(1)	1.74		3(0)	2		1(1)	1.70	
	4 weeks	1(0)	1.26		3(1)	1.67		1(0)	1.30	
C4	Baseline	3(0)	3	<0.001	3(0)	2.04	0.135	3(0)	3	<0.001
	2 weeks	1(1)	1.65		3(0)	2.04		1(0)	1.61	
	4 weeks	1(0)	1.35		3(0)	1.91		1(0)	1.39	
Migraine-specific quality of life										
Domain 1	Baseline	5(0)	3.50	<0.001	5(0)	3.17	<0.001	5(1)	3.50	<0.001
	2 weeks	3(1)	1.96		4(1)	2.22		3(1)	1.98	
	4 weeks	2(1)	1.04		4(0)	1.43		2(1)	1.02	
Domain 2	Baseline	5(1)	3.50	<0.001	4(2)	3.85	<0.001	5(2)	2.50	<0.001
	2 weeks	3(2)	1.85		4(1)	2.33		3(1)	1.91	
	4 weeks	2(2)	1.15		4(1)	1.98		2(1)	1.09	
Domain 3	Baseline	4(1)	3.50	<0.001	5(1)	2.70	<0.001	4(2)	3.50	<0.001
	2 weeks	3(1)	1.98		5(1)	2.70		2(1)	1.87	
	4 weeks	1(1)	1.04		4(2)	1.91		2(1)	1.13	
MSQ total	Baseline	5(1)	3.50	<0.001	5(1)	2.96	<0.001	5(1)	3.50	<0.001
	2 weeks	2(1)	1.91		4(1)	2.50		3(1)	1.98	
	4 weeks	2(1)	1.09		4(1)	1.59		2(1)	1.02	

NPS: Numeric pain scale, A1: Interference of pain, A2: Support received by others, A3: Pain severity, A4: Control perception, A5: Effective distress, B1: Punishing response, B2: Solicitous response, B3: Distracting response, C1: Household chores, C2: Outdoor work, C3: Activities away from home, C4: Social activities, Domain 1: Restrictive, Domain 2: Preventive, Domain 3: Emotional function, MSQ: Migraine-specific quality of life.

## Data Availability

The dataset(s) supporting the conclusions of this article is available in the Figshare repository, 10.6084/m9.figshare.27261753.

## References

[1] Ferrari M.D., Goadsby P.J., Burstein R., Kurth T., Ayata C., Charles A., Ashina M., van den Maagdenberg A.M.J.M., Dodick D.W. (2022). Migraine (Primer). Nat. Rev. Dis. Primers.

[2] Martin L.F., Patwardhan A.M., Jain S.V., Salloum M.M., Freeman J., Khanna R., Gannala P., Goel V., Jones-MacFarland F.N., Killgore W.D. (2021). Evaluation of green light exposure on headache frequency and quality of life in migraine patients: A preliminary one-way cross-over clinical trial. Cephalalgia.

[3] Puledda F., Silva E.M., Suwanlaong K., Goadsby P.J. (2023). Migraine: From pathophysiology to treatment. J. Neurol..

[4] Woldeamanuel Y.W., Cowan R.P. (2017). Migraine affects 1 in 10 people worldwide featuring recent rise: A systematic review and meta-analysis of community-based studies involving 6 million participants. J. Neurol. Sci..

[5] Hussain G., Rasul A., Anwar H., Sohail M.U., Kamran S.K.S., Baig S.M., Shabbir A. (2017). Epidemiological data of neurological disorders in Pakistan and neighboring countries: A review. Pak. J. Neurol. Sci. (PJNS).

[6] Qubty W., Patniyot I. (2020). Migraine pathophysiology. Pediatr. Neurol..

[7] Lipton R.B., Fanning K.M., Buse D.C., Martin V.T., Reed M.L., Adams A.M., Goadsby P.J. (2018). Identifying natural subgroups of migraine based on comorbidity and concomitant condition profiles: Results of the chronic migraine epidemiology and outcomes (CaMEO) study. Headache J. Head Face Pain.

[8] Diener H.-C., Solbach K., Holle D., Gaul C. (2015). Integrated care for chronic migraine patients: Epidemiology, burden, diagnosis and treatment options. Clin. Med..

[9] Eigenbrodt A.K., Ashina H., Khan S., Diener H.-C., Mitsikostas D.D., Sinclair A.J., Pozo-Rosich P., Martelletti P., Ducros A., Lantéri-Minet M. (2021). Diagnosis and management of migraine in ten steps. Nat. Rev. Neurol..

[10] Munksgaard S.B., Jensen R.H. (2014). Medication overuse headache. Headache J. Head Face Pain.

[11] Lipton R.B., Munjal S., Buse D.C., Alam A., Fanning K.M., Reed M.L., Schwedt T.J., Dodick D.W. (2019). Unmet acute treatment needs from the 2017 migraine in America symptoms and treatment study. Headache J. Head Face Pain.

[12] Puledda F., Shields K. (2018). Non-pharmacological approaches for migraine. Neurotherapeutics.

[13] Lang N., Nitsche M.A., Paulus W., Rothwell J.C., Lemon R.N. (2004). Effects of transcranial direct current stimulation over the human motor cortex on corticospinal and transcallosal excitability. Exp. Brain Res..

[14] Sauseng P., Klimesch W., Gerloff C., Hummel F.C. (2009). Spontaneous locally restricted EEG alpha activity determines cortical excitability in the motor cortex. Neuropsychologia.

[15] Yuan H., Perdoni C., Yang L., He B. (2011). Differential electrophysiological coupling for positive and negative BOLD responses during unilateral hand movements. J. Neurosci..

[16] Cambiaghi M., Velikova S., Gonzalez-Rosa J.J., Cursi M., Comi G., Leocani L. (2010). Brain transcranial direct current stimulation modulates motor excitability in mice. Eur. J. Neurosci..

[17] DaSilva A.F., Datta A., Swami J., Kim D.J., Patil P.G., Bikson M. (2022). The Concept, Development, and Application of a Home-Based High-Definition tDCS for Bilateral Motor Cortex Modulation in Migraine and Pain. Front. Pain Res..

[18] Brunoni A.R., Moffa A.H., Fregni F., Palm U., Padberg F., Blumberger D.M., Daskalakis Z.J., Bennabi D., Haffen E., Alonzo A. (2016). Transcranial direct current stimulation for acute major depressive episodes: Meta-analysis of individual patient data. Br. J. Psychiatry.

[19] Vandewalle G., Maquet P., Dijk D.J. (2009). Light as a modulator of cognitive brain function. Trends Cogn. Sci..

[20] Vandewalle G., Balteau E., Phillips C., Degueldre C., Moreau V., Sterpenich V., Albouy G., Darsaud A., Desseilles M., Dang-Vu T.T. (2006). Daytime light exposure dynamically enhances brain responses. Curr. Biol..

[21] Argilés M., Sunyer-Grau B., Arteche-Fernandez S., Peña-Gómez C. (2022). Functional connectivity of brain networks with three monochromatic wavelengths: A pilot study using resting-state functional magnetic resonance imaging. Sci. Rep..

[22] Hou T.W., Yang C.C., Lai T.H., Wu Y.H., Yang C.P. (2024). Light Therapy in Chronic Migraine. Curr. Pain Headache Rep..

[23] Nagata E., Takao M., Toriumi H., Suzuki M., Fujii N., Kohara S., Tsuda A., Nakayama T., Kadokura A., Hadano M. (2024). Hypersensitivity of Intrinsically Photosensitive Retinal Ganglion Cells in Migraine Induces Cortical Spreading Depression. Int. J. Mol. Sci..

[24] Gerasimova E., Burkhanova G., Chernova K., Zakharov A., Enikeev D., Khaertdinov N., Giniatullin R., Sitdikova G. (2021). Hyperhomocysteinemia increases susceptibility to cortical spreading depression associated with photophobia, mechanical allodynia, and anxiety in rats. Behav. Brain Res..

[25] Cheng K. (2023). A Descending Pain Modulation Pathway in Green Light-Induced Antinociception. Ph.D. Dissertation.

[26] Faul F., Erdfelder E., Lang A.G., Buchner A. (2007). G* Power 3: A flexible statistical power analysis program for the social, behavioral, and biomedical sciences. Behav. Res. Methods.

[27] Rocha S., Melo L., Boudoux C., Foerster Á., Araújo D., Monte-Silva K. (2015). Transcranial direct current stimulation in the prophylactic treatment of migraine based on interictal visual cortex excitability abnormalities: A pilot randomized controlled trial. J. Neurol. Sci..

[28] Peng K.-P., Wang S.-J. (2012). Migraine diagnosis: Screening items, instruments, and scales. Acta Anaesthesiol. Taiwanica.

[29] Adeboye A., Hart R., Senapathi S.H., Ali N., Holman L., Thomas H.W. (2021). Assessment of functional pain score by comparing to traditional pain scores. Cureus.

[30] Sohn E.H., Kim B.J. (2021). Clinical scale for neuropathic pain. J. Korean Neurol. Assoc..

[31] Peipert A., Engel E., Ehrlich-Jones L. (2018). Measurement characteristics and clinical utility of the West Haven-Yale Multidimensional Pain Inventory in a chronic pain population. Arch. Phys. Med. Rehabil..

[32] Gerdle B., Cervin M., Rivano Fischer M., Ringqvist Å. (2021). Outcomes of interdisciplinary pain rehabilitation across subgroups of the multidimensional pain inventory–a study from the Swedish quality Registry for pain rehabilitation. Pain Pract..

[33] Shibata M., Nakamura T., Ozeki A., Ueda K., Nichols R.M. (2020). Migraine-Specific Quality-of-Life Questionnaire (MSQ) version 2.1 score improvement in Japanese patients with episodic migraine by galcanezumab treatment: Japan phase 2 study. J. Pain Res..

[34] Wells R.E., O’Connell N., Pierce C.R., Estave P., Penzien D.B., Loder E., Zeidan F., Houle T.T. (2021). Effectiveness of mindfulness meditation vs headache education for adults with migraine: A randomized clinical trial. JAMA Intern. Med..

[35] Dalla Volta G., Marceglia S., Zavarise P., Antonaci F. (2020). Cathodal tDCS guided by thermography as adjunctive therapy in chronic migraine patients: A sham-controlled pilot study. Front. Neurol..

[36] Andrade S.M., de Brito Aranha R.E.L., de Oliveira E.A., de Mendonça C.T.P.L., Martins W.K.N., Alves N.T., Fernández-Calvo B. (2017). Transcranial direct current stimulation over the primary motor vs prefrontal cortex in refractory chronic migraine: A pilot randomized controlled trial. J. Neurol. Sci..

[37] Şirin T.C., Aksu S., Bayir B.R.H., Ulukan Ç., Karamürsel S., Kurt A., Baykan B. (2021). Is allodynia a determinant factor in the effectiveness of transcranial direct current stimulation in the prophylaxis of migraine?. Neuromodulation Technol. Neural Interface.

[38] DaSilva A.F., Mendonca M.E., Zaghi S., Lopes M., DDS M.F.D., Spierings E.L., Bajwa Z., Datta A., Bikson M., Fregni F. (2012). tDCS-induced analgesia and electrical fields in pain-related neural networks in chronic migraine. Headache J. Head Face Pain.

[39] Auvichayapat P., Janyacharoen T., Rotenberg A., Tiamkao S., Krisanaprakornkit T., Sinawat S., Punjaruk W., Thinkhamrop B., Auvichayapat N. (2012). Migraine prophylaxis by anodal transcranial direct current stimulation, a randomized, placebo-controlled trial. J. Med. Assoc. Thai..

[40] La Touche R., Pérez J.J.F., Acosta A.P., Campodónico L.G., García S.M., Juárez D.A., García B.S., Angulo-Díaz-Parreño S., Cuenca-Martínez F., Suso-Martí L. (2020). Is aerobic exercise helpful in patients with migraine? A systematic review and meta-analysis. Scand. J. Med. Sci. Sports.

[41] Ahdab R., Mansour A.G., Khazen G., El-Khoury C., Sabbouh T.M., Salem M., Yamak W., Ayache S.S., Riachi N. (2019). Cathodal transcranial direct current stimulation of the occipital cortex in episodic migraine: A randomized sham-controlled crossover study. J. Clin. Med..

